# Peptide modification results in the formation of a dimer with a 60-fold enhanced antimicrobial activity

**DOI:** 10.1371/journal.pone.0173783

**Published:** 2017-03-15

**Authors:** Amal Thamri, Myriam Létourneau, Alex Djoboulian, David Chatenet, Eric Déziel, Annie Castonguay, Jonathan Perreault

**Affiliations:** INRS - Institut Armand-Frappier, Université du Québec, Laval, Québec, Canada; Laurentian, CANADA

## Abstract

Cationic antimicrobial peptides (CAMPs) occur naturally in numerous organisms and are considered as a class of antibiotics with promising potential against multi-resistant bacteria. Herein, we report a strategy that can lead to the discovery of novel small CAMPs with greatly enhanced antimicrobial activity and retained antibiofilm potential. We geared our efforts towards *i*) the N-terminal cysteine functionalization of a previously reported small synthetic cationic peptide (peptide 1037, KRFRIRVRV-NH_2_), *ii*) its dimerization through a disulfide bond, and *iii*) a preliminary antimicrobial activity assessment of the newly prepared dimer against *Pseudomonas aeruginosa* and *Burkholderia cenocepacia*, pathogens responsible for the formation of biofilms in lungs of individuals with cystic fibrosis. This dimer is of high interest as it does not only show greatly enhanced bacterial growth inhibition properties compared to its pep1037 precursor (up to 60 times), but importantly, also displays antibiofilm potential at sub-MICs. Our results suggest that the reported dimer holds promise for its use in future adjunctive therapy, in combination with clinically-relevant antibiotics.

## Introduction

Antibiotic resistance is a serious and growing phenomenon, as well as a primary public health concern [1, 2]. Unfortunately, antibiotics are no longer the magic bullets that they once were. New resistance mechanisms have emerged, making generations of antibiotics virtually ineffective, resulting in prolonged illness, greater risk of death and higher costs. Thus, development of new antibiotics and other novel strategies are critically needed. Biofilm-associated bacteria possess 10–1,000 fold greater resistance to antibiotic treatment compared to freely-floating, planktonic cells, making established biofilm infections especially difficult to eradicate [3, 4]. The biofilm mode of growth is a strategy used by microorganisms to survive unfavorable environmental conditions. An estimated 80% of all bacterial infections involve a biofilm component [5]. For instance, the severe antibiotic resistance of *Pseudomonas aeruginosa* and *Burkholderia cenocepacia* in lungs of Cystic Fibrosis (CF) patients has been associated with the formation of drug resistant biofilms [6, 7]. Bacteria benefit from the accumulation of thick and sticky mucus to colonize the lung tissue and airways in a multi-resistant biofilm, which results in respiratory failure and mortality [7]. To date, no antimicrobial that targets bacterial biofilm(s) has been clinically approved. Thus, there is a great interest in the development of antimicrobials that inhibit bacterial growth, and also prevent biofilm formation and/or promote established biofilm dissolution.

Cationic antimicrobial peptides (CAMPs) are a class of antibiotics with promising potential against multi-resistant bacteria. Various CAMPs with a broad spectrum activity were isolated from invertebrates, insects, microorganisms, plants, fish, amphibians, mammals and humans [8]. Attracted to the anionic phospholipid head, CAMPs establish strong nonspecific, hydrophobic and electrostatic interactions with the bacterial cytoplasmic membrane [9]. Although their therapeutic potential seems to be restricted, as it is the case for many other types of antimicrobials, by the difficulty to find a proper balance between critical parameters such as hydrophobicity, charge, length, amphiphilicity and structure, CAMPs have the advantage to be small and easily tunable. Still, their use remains restricted by their intrinsic characteristics, notably their toxicity to mammalian cells, limited tissue distribution and proteolytic degradation in the blood [10]. Among successful examples, the synthetic protegrin analog IB-367 is part of an aerosol formulation used after chemotherapy [11]. Although their mechanism of action is not completely established, it is recognized that CAMPs can interfere with different stages of bacterial biofilm formation. Initially, CAMPs prevent cell adhesion to the surface *via* electrostatic bonds with the membrane [12]. They are also capable of preventing biofilm maturation by acting on the molecular signals involved in quorum sensing [13]. A library of small synthetic CAMPs able to inhibit biofilm formation and promote biofilm degradation can be found in the BaAMPs database (biofilm active antimicrobial peptides database) [14].

The discovery of naturally occurring dimeric peptides with marked antimicrobial activity encouraged scientists to explore dimerization, including disulfide bridging and lactamization, as a means to confer to CAMPs many properties that may enhance their therapeutic potential as compared to their monomers [15, 16]. For instance, the Defr1 dimeric defensin peptide, containing a natural cysteine residue, has an activity against *P*. *aeruginosa* PAO1 (6 μg/mL) approximately 9-fold greater than its 34-residue monomer counterpart (50 μg/mL) [17]. Also, compared to its monomeric form, VG16KRKP dimer displays a 10-fold decrease of its MIC against *E*. *coli* and more than a 4-fold decrease against *P*. *aeruginosa* and *K*. *pneumoniae* [18]. The improved antibacterial activity was correlated with an increase in hydrophobicity and cationicity of its surface area, which enhances LPS binding and neutralization [16]. Interestingly, very few studies reported the effect of CAMP dimerization through cysteine disulfide bridge formation, especially for N-terminus cysteine-containing peptides [18].

Herein, we report the N-terminal functionalization of a small synthetic cationic peptide with a cysteine, its dimerization through a disulfide bond, and a preliminary antimicrobial activity assessment of the newly prepared dimer against two strains each of *P*. *aeruginosa* and *B*. *cenocepacia*. For this study, the synthetic 9-mer cationic peptide **pep1037** (KRFRIRVRV-NH_2_) was selected [19], due to its antimicrobial potential against *P*. *aeruginosa* and *B*. *cenocepacia* and its considerable antibiofilm activity. We report that such a strategy leads to the discovery of a small CAMP with greatly enhanced antimicrobial activity and retained antibiofilm potential.

## Materials and methods

### Peptide synthesis/modification

**Fig 1:**
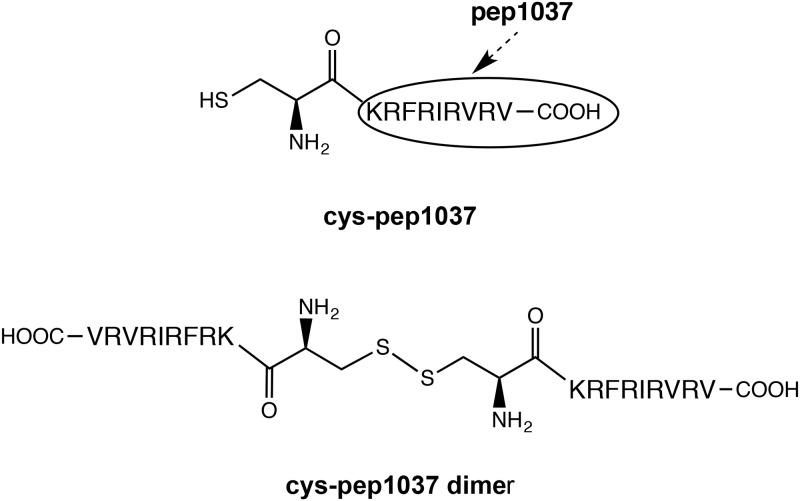
Structure of cys-pep1037 and its dimeric form.

**Pep1037** and **cys-pep1037** were synthesized manually using a standard solid phase peptide synthesis approach with Fmoc chemistry (Fig 1). Couplings of the protected amino acids were mediated by benzotriazol-1-yl-oxy-tris-(dimethylamino)phosphonium hexafluorophosphate (BOP; 3 equiv) and N,N'-diisopropylethylamine (DIPEA; 6 equiv) in N,N-dimethylformamide (DMF) at room temperature for 0.5–1 h. Coupling efficiency was monitored with the qualitative ninhydrin test and a 3-equivalent excess of the protected amino acids based on the original substitution of the Fmoc Rink-amide resin (0.62 mmol/g). Fmoc removal was achieved with 20% piperidine in DMF at room temperature for 10 minutes. Peptides were cleaved from the resin support with simultaneous side chain deprotection by treatment with TFA containing 1,2-ethanedithiol (2.5%), water (5%), triisopropylsilane (1%), thioanisole (5%) and phenol (5%) for 4 h at room temperature. After cleavage, the resin was removed by filtration, the filtrate was concentrated and peptides were precipitated using cold diethyl ether. Crude peptides were then solubilized in water and lyophilized prior to their purification using preparative RP-HPLC. **Cys-pep1037 dimer** was isolated by reversed phase HPLC from a lyophilized sample of crude **cys-pep1037**. **Mal-cys-pep1037** was prepared and isolated using the following procedure: **cys-pep1037** was dissolved to a final concentration of 1mg/mL in fresh PBS (phosphate buffer saline, pH = 7), and an excess of tris-carboxyethylphosphine (TCEP) reagent (10 equiv) was added (to prevent the formation of disulfide bonds). The mixture was stirred at room temperature for 20 min, an excess of maleimide (2 equiv) was added and was allowed to react for 4 h. Peptide **mal-cys-pep1037** was isolated from the mixture by RP-HPLC.

All crude lyophilized peptides were purified using a preparative reversed-phase HPLC (RP-HPLC) protocol using a linear gradient from eluent A to B with 25% B per 2 min increments (Eluent A = H_2_O, 0.1% TFA, Eluent B = CH_3_CN/40% A, 0.1% TFA) and a C18 (column Phenomenex, Jupiter^®^, 15μm, 10 x 300 mm, 4 mL/min, absorbance measured at 220nm). Homogeneity of purified fractions was assessed by RP-HPLC and MALDI-TOF mass spectrometry in linear mode using α-cyanohydroxycinnamic acid (matrix). Fractions containing the desired product were pooled and lyophilized. Overall, RP-HPLC analysis of each analog revealed that the purity of all peptides was ≥ 97%. For each peptide, the main peak observed by mass spectrometry (S1 Fig) was in agreement with the theoretical mass values (**pep1037**: *m/z* ([M+H]^+^) 1229.3; **cys-pep1037**: *m/z* ([M+H]^+^) 1331.8; **mal-cys-pep1037**: *m/z* ([M+H]^+^) 1429.7; **cys-pep1037 dimer**: *m/z* ([M+H]^+^) 2662.5).

#### Bacterial strains and growth conditions

*P*. *aeruginosa* ATCC 27853 (blood culture isolate, American Type Culture Collection, Manassas, USA), *P*. *aeruginosa* ATCC 15442 (animal room water bottle isolate), *B*. *cenocepacia* K56-2 (CF patient isolate) and *B*. *cenocepacia* J2315 (multidrug-resistant CF patient isolate) were used. Bacteria were subcultured on TSA (tryptic soy agar, BD Difco^™^ Dehydrated Culture Media, Fisher Scientific) plates from frozen stocks. Inocula was prepared in TSB (typtic soy broth, BD Difco^™^ Dehydrated Culture Media, fisher scientific) in overnight cultures at 37°C with shaking at 200 rpm.

#### Minimum Inhibitory Concentration (MIC) determination

The broth microdilution method (with minor modifications for cationic peptides) was performed according to the guidelines of "*Methods for Dilution Antimicrobial Susceptibility Tests for Bacteria That Grow Aerobically; Approved Standard-Ninth Edition*" developed by the Clinical Laboratory Standards Institute’s committee [20]. Peptides were dissolved in water and stored in glass vials. Different peptide concentrations were prepared in 100 μL of cationic adjusted (25 mg/L CaCl_2_ and 12.5 mg/L MgCl_2_) MHB (Mueller Hinton broth, BD Difco^™^ Dehydrated Culture Media, Fisher Scientific) and added to sterile 96-well polypropylene microtiter plates. Each well was inoculated to a final concentration of 5 × 10^4^ CFU (50 μL of MHB inocula suspension prepared from colonies grown on MHA) [21]. Plates were incubated at 37°C under static conditions for 18 h and 24 h for *P*. *aeruginosa* and *B*. *cenocepacia*, respectively, and absorbance was read at 600 nm using a Cytation 3-cell imaging plate reader. The MIC was defined as the lowest concentration of peptide at which no growth was observed. All experiments were done at least in triplicate.

#### Biofilm inhibition assay

The ability of **pep1037**, **cys-pep1037 dimer** and **mal-cys-pep1037** to prevent biofilm formation was evaluated by a method previously described [22, 23] with the following modifications. *P*. *aeruginosa* and *B*. *cenocepacia* cells (1 × 10^5^ CFU) were grown in 96-well polypropylene microtiter plates (BD falcon # 353912) in 200 μL final volume of TSB at 37°C for 24 h in the presence (2.5 μg/mL) or not of the peptides. Optical density (OD) of the cultures at 600 nm was determined after inoculation. As a measure of the planktonic bacterial growth, OD was again measured after 24 h of incubation, prior to biofilm staining. The planktonic suspension was gently aspirated using pipettes. Wells were then washed by 200 μL PBS and shaked out by turning the plate over. Biofilm cells were fixed by 200 μL of 99% methanol per well for 15 min. Then, methanol was aspirated and plates were air dried overnight. Biofilm production was measured using the crystal violet (CV, 1%) stain technique [23]. Briefly, excess CV was rinsed three times with water and plates were air-dried overnight. Stain was solubilized in 250 μL of 30% glacial acetic acid and quantified using a plate reader at 570 nm. All experiments were done in six replicates.

#### Biofilm disintegration assay

The pre-formed biofilm assay was performed as previously described [24]. *P*. *aeruginosa* ATCC 27853 or ATCC 15442 (1 × 10^5^ CFU) were incubated under static conditions in 200 μL of TSB for 24 h at 37°C. TSB media was then aspirated and the established biofilm was treated with various concentrations of **pep1037**, **cys-pep1037 dimer** and **mal-cys-pep1037** solutions prepared in fresh TSB. The microtiter plate was incubated for 24 h, at 37°C. Biofilm production was measured using the crystal violet stain technique as described above. All experiments were done in six replicates.

## Results and discussion

To assess the impact of **pep1037** dimerization on bacterial growth and biofilm formation/degradation, a cysteine residue was introduced at its N-terminus, **cys-pep1037**. As reported for other cysteine containing peptides [25–28], dimerization occurs spontaneously during the lyophilization/purification step following the synthesis of **cys-pep1037**. Both peptides, **cys-pep1037** and **cys-pep1037 dimer**, were isolated using RP-HPLC (Fig 1).

### Inhibition of bacterial growth

The ability of **cys-pep1037 dimer** to inhibit the growth of *P*. *aeruginosa* was evaluated by determining its minimum inhibitory concentration (MIC) against two different strains of *P*. *aeruginosa*, notably ATCC 27853 and ATCC 15442, and results were compared to the MIC of native **pep1037** against the same strains. MIC values determined for **pep1037** were in agreement with previously reported MIC values of the same peptide against two Gram-negative pathogens, *P*. *aeruginosa* (PAO1 and PA14) and *B*. *cenocepacia* (4813), from 304 to > 608 μg/mL, respectively [19]. Interestingly, **cys-pep1037 dimer** was found to display a 30 to 60 times lower MIC than **pep1037** against *P*. *aeruginosa* ATCC 27853 and ATCC 15442, respectively (Table 1). The MIC of pure **cys-pep1037** could not be assessed due the presence of variable amounts of the corresponding dimer in the stock solutions used, but nevertheless, the MIC of this peptide mixture was found to be much lower than that of **pep1037**, but 4–8 times higher than that of **cys-pep1037 dimer** alone. This result strongly suggests that the antimicrobial activity of this peptide mixture is due to the presence of the dimer, although one cannot rule out the possible additional contribution of **cys-pep1037**. It is important to note that the dimer to monomer ratio might increase over time during MIC determination experiments, as considerable amounts of dimer were observed when solutions of **cys-pep1037** (40 μg/mL) were incubated in culture medium for 1 h.

**Table 1:** Inhibitory effect of the peptides on bacterial growth^a^.

Bacteria (strain)	MIC (μg/mL)			
	pep1037^b^	cys-pep1037^c^	cys-pep1037 dimer	mal-cys-pep1037
*P*. *aeruginosa* (ATCC 27853)	307	40	10	357
*P*. *aeruginosa* (ATCC 15442)	307	40	5	357
*B*. *cenocepacia* (J2315)	> 614	332	332	> 614
*B*. *cenocepacia* (K56-2)	> 614	332	332	> 614

^a^Identical results were obtained for all replicates.

^b^Additional species and strains were also tested (see S1 Table).

^c^Different stock solutions of **cys-pep1037** (13 mg/mL) used for this experiment contained variable amounts of **cys-pep1037 dimer**.

**Fig 2:**
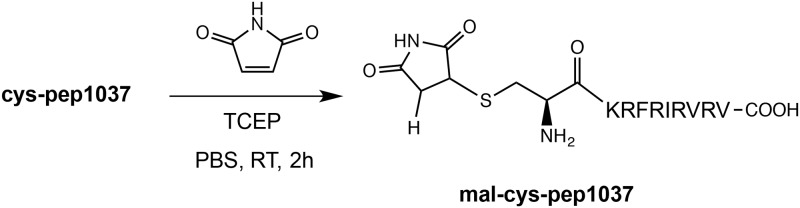
Formation of mal-cys-pep1037 *via* a thiolene reaction between cys-pep1037 and maleimide.

To prevent **cys-pep1037** from undergoing dimerization, its thiol functionality was reacted with maleimide (Fig 2). **Mal-cys-pep1037** displayed a lower antimicrobial activity against both strains of *P*. *aeruginosa* compared to its precursor, **cys-pep1037**, but an activity equivalent to that of **pep1037**. This again supports the previously mentioned hypothesis that the antimicrobial activity of the **cys-pep1037** monomer/dimer mixture might be due to the presence of **cys-pep1037 dimer**, without completely ruling out an effect from the presence of an additional cysteine residue at the N-terminal position of **pep1037**. Indeed, bringing modifications at the N-terminal position of a peptide can have a drastic impact on its antimicrobial activity [29–31].

All peptides were also tested against two strains of *B*. *cenocepacia*, bacteria that also play a role in CF lung infection. Their antibacterial potential against both strains was found to be at least two times higher than the one of **pep1037** (332 μg/mL compared to > 614 μg/mL), but in contrast to what was noted for both *P*. *aeruginosa* strains, **cys-pep1037 dimer** did not show an enhancement in antibacterial activity compared to the **cys-pep1037** monomer / dimer mixture (Table 1). Dimerization of **cys-pep1037** from the monomer / dimer mixture in cultures could explain these results. This is further supported by the loss of **cys-pep1037** enhanced antibacterial effect (Table 1) upon reaction with maleimide, likely due to the inability of **mal-cys-pep1037** to dimerize.

### Biofilm inhibition and disintegration

**Fig 3:**
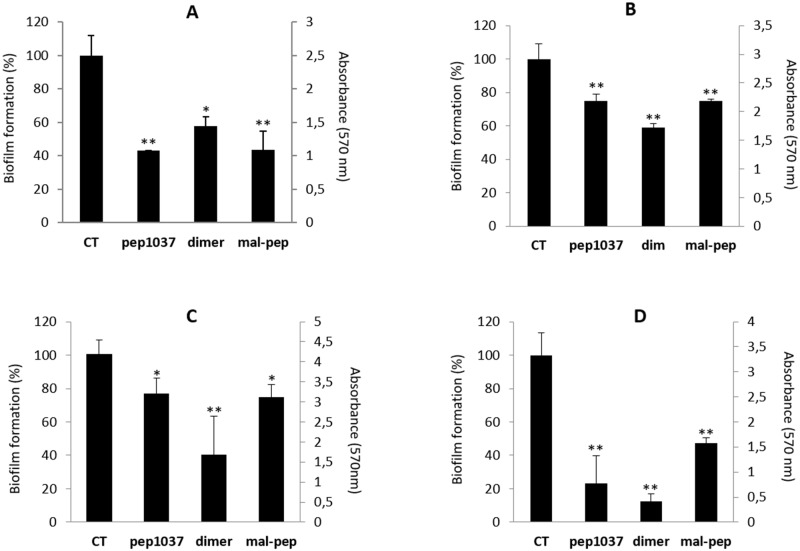
Inhibiting effect of 10 μg/mL of pep1037, cys-pep1037 dimer and mal-cys-pep1037 on the formation of *P*. *aeruginosa* ATCC 15442 (A), *P*. *aeruginosa* ATCC 27853 (B), *B*. *cenocepacia* K56-2 (C) and *B*. *cenocepacia* J2315 (D) biofilms. Error bars indicate the standard deviation of six replicates, and statistical significance was determined using one-way ANOVA (*, P < 0.01; **, P < 0.001 versus the control, denoted CT).

**Fig 4:**
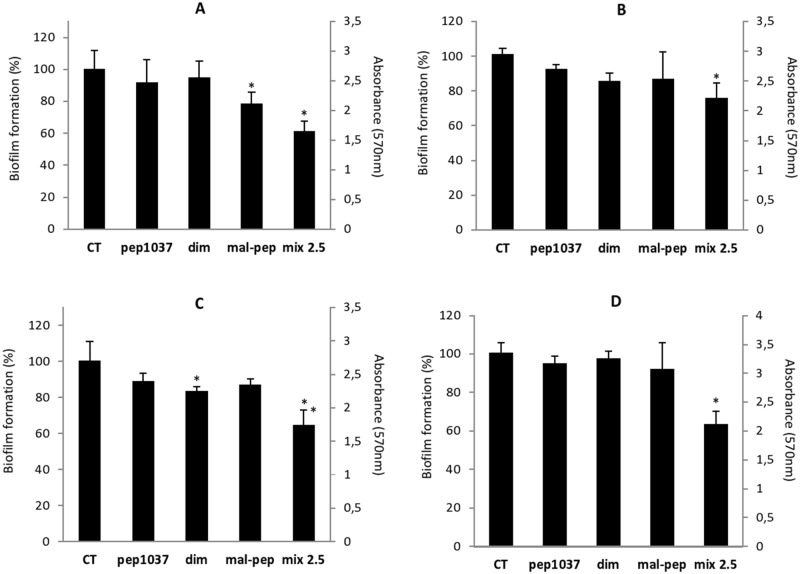
Inhibiting effect of 2.5 μg/mL of pep1037, cys-pep1037 dimer, mal-cys-pep1037 and a combination of pep1037 and cys-pep1037 dimer (denoted mix 2.5) on the formation of *P*. *aeruginosa* ATCC 15442 (A), *P*. *aeruginosa* ATCC 27853 (B), *B*. *cenocepacia* K56-2 (C) and *B*. *cenocepacia* J2315 (D) biofilms. Error bars indicate the standard deviation of six replicates, and statistical significance was determined using one-way ANOVA (*, *P* < 0.01 versus the control, denoted CT).

To further explore the potential of **cys-pep1037 dimer**, we investigated whether the antibiofilm ability of its monomeric precursor **pep1037** could be retained. The inhibitory effect of **cys-pep1037 dimer** on the formation of *P*. *aeruginosa* and *B*. *cenocepacia* biofilms was then assessed using the crystal violet assay (Figs 3 and 4). For comparison purposes, the inhibitory effect of **mal-cys-pep1037** was also evaluated and results were compared to the ones obtained for **pep1037** (which were found to be in agreement with previous reports) [19]. From these experiments, we conclude that *i*) the modification of **pep1037** with a maleimide-protected cysteine at the N-terminal position (**mal-cys-pep1037**), and *ii)* the dimerization of its cysteine N-terminal adduct (**cys-pep1037 dimer**) do not lead to an overall loss in its ability to prevent biofilm formation. More specifically, at a concentration of 10 μg/mL, all peptides displayed a considerable effect on the formation of the four biofilms studied after 24h (Fig 3). A particularly pronounced effect of the peptides was noted against the *B*. *cenocepacia* J2315 biofilm (Fig 3D), 10 μg/mL being at least 30 times lower than their respective MIC (Table 1). Many CAMPs were reported to display a biofilm formation inhibitory effect at a concentration much lower than their MIC [29, 32, 33], but very few of these CAMPs were found to display a significant antimicrobial activity against planktonic bacteria [34]. Since the inhibitory effect observed for **cys-pep1037 dimer** on the formation of both *P*. *aeruginosa* strains could simply be due to its high potential to inhibit planktonic bacterial growth (MIC = 5–10, Table 1), the same experiment was also performed for all peptides at a concentration of 2.5 μg/mL. No significant inhibitory effect was observed on the formation of the four biofilms for any of the peptides when a sub-MIC concentration of 2.5 μg/mL was used (Fig 4) but interestingly, the combination of **cys-pep1037 dimer** (2.5 μg/mL) and **pep1037** (2.5 μg/mL) led to a considerable inhibitory effect in the range of 60–80% (Fig 4). This highlights the interest of further studying the potential use of sub-MIC concentrations of this dimer in adjunctive therapy.

**Fig 5:**
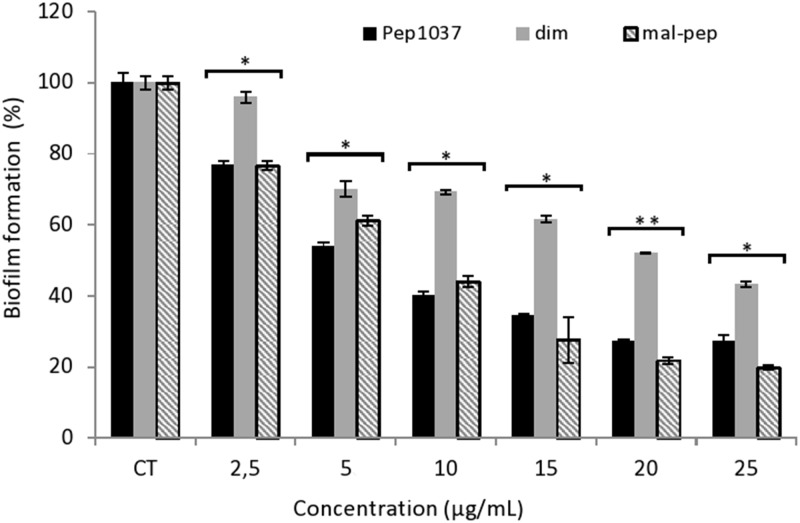
Effect of pep1037, cys-pep1037 dimer and mal-cys-pep1037 on the degradation of an established biofilm of a *P*. *aeruginosa* ATCC 27853 after 24 h. Error bars indicate the standard deviation of six replicates, and statistical significance was determined using ANOVA (*, *P* < 0.01; **, *P* < 0.001 versus the control, denoted CT).

The biofilm disintegration potential of **pep1037**, **cys-pep1037 dimer** and **mal-cys-pep1037** was also assessed on a *P*. *aeruginosa* (ATCC 27853) preformed biofilm. All three peptides were found to have a considerable potential to disturb the established biofilm studied (Fig 5). A concentration of 10 μg/mL of **pep1037** or **mal-cys-pep1037** is sufficient to reduce by 56–60% the established monospecies biofilm, and there is no significant difference between the effect of both these peptides on biofilm degradation. Some CAMPs were previously reported to induce the dispersion of viable cells of mature biofilms [35]. Interestingly, for all concentrations studied, the established biofilm is reduced to a lesser extent by **cys-pep1037 dimer** than when treated with **pep1037** or **mal-cys-pep1037** (Fig 5). The effect of **cys-pep1037 dimer** on mature biofilm might not only be affected by its interaction with organisms in the *P*. *aeruginosa* biofilm but might also be strongly related to its ability to diffuse into the biofilm, as suggested for previously reported peptides [36]. The ability of cationic antibiotics to diffuse into biofilms is negatively influenced by an increase in molecular weight [37], and electrostatic interactions of cationic peptides with negatively charged biofilm matrix can delay CAMPs penetration [38]. These factors might explain why **cys-pep1037 dimer** is less active than its monomeric precursor **pep1037** on the pre-formed biofilm tested. Nevertheless, the potential of the dimeric form of **cys-pep1037** to degrade an established *P*. *aeruginosa* biofilm is retained at sub-MIC concentrations.

## Conclusion

We report here the N-terminal functionalization of a small synthetic cationic peptide with a cysteine, leading to the spontaneous formation of a dimer with a 30 to 60-fold enhancement in antimicrobial activity compared to its precursor. This work demonstrates that this strategy can potentially lead to the discovery of relatively small CAMPs with a greatly enhanced antimicrobial activity through a simple approach. Importantly, this can be achieved while retaining considerable activity against mature biofilms at sub-MICs. *P*. *aeruginosa* and *B*. *cenocepacia* are important pathogens responsible for various opportunistic infections, including the morbidity and mortality in CF patients *via* the formation of biofilms in lungs. These results suggest that **cys-pep1037 dimer** holds promise for its use in adjunctive therapy and future research may demonstrate potential for its use in combination with clinically-relevant antibiotics [39]. This study also opens the door to the improvement of small CAMPs *via* cysteine disulfide dimerization at the N-terminus, which has so far been unexplored.

## Supporting information

S1 Table Bacterial growth inhibitory effect of the peptides. (DOCX)

S1 Fig Mass spectral data for all peptides (MALDI-TOF mass spectrometry in linear mode using α-cyanohydroxycinnamic acid as matrix). (DOCX)

## References

[1] Leclercq R. Mondialisation de la résistance bactérienne aux antibiotiques. Med Sci (Paris). 2008;24:32–7.

[2] Marston HD, Dixon DM, Knisely JM, Palmore TN, Fauci AS. Antimicrobial Resistance. JAMA. 2016;316(11):1193–204. 10.1001/jama.2016.11764 27654605

[3] Davies D. Understanding biofilm resistance to antibacterial agents. Nat Rev Drug Discov. 2003;2(2):114–22. 10.1038/nrd1008 12563302

[4] Schwartz T, Kohnen W, Jansen B, Obst U. Detection of antibiotic-resistant bacteria and their resistance genes in wastewater, surface water, and drinking water biofilms. FEMS Microbiol Ecol. 2003;43(3):325–35. 10.1111/j.1574-6941.2003.tb01073.x 19719664

[5] Romling U, Balsalobre C. Biofilm infections, their resilience to therapy and innovative treatment strategies. J Intern Med. 2012;272(6):541–61. 10.1111/joim.12004 23025745

[6] Moreau-Marquis S, Stanton BA, O’Toole GA. Pseudomonas aeruginosa biofilm formation in the cystic fibrosis airway. Pulmonary Pharmacology & Therapeutics. 2008;21(4):595–9. 18234534 10.1016/j.pupt.2007.12.001 PMC2542406

[7] Tomlin KL, Coll OP, Ceri H. Interspecies biofilms of Pseudomonas aeruginosa and Burkholderia cepacia. Can J Microbiol. 2001;47(10):949–54. 11718549

[8] Wang G, Li X, Wang Z. APD3: the antimicrobial peptide database as a tool for research and education. Nucleic Acids Res. 2016;44(Database issue):D1087–93. 26602694 10.1093/nar/gkv1278 PMC4702905

[9] Godballe T, Nilsson LL, Petersen PD, Jenssen H. Antimicrobial beta-peptides and alpha-peptoids. Chem Biol Drug Des. 2011;77(2):107–16. 10.1111/j.1747-0285.2010.01067.x 21266014

[10] Bahar AA, Ren D. Antimicrobial peptides. Pharmaceuticals (Basel). 2013;6(12):1543–75. 24287494 10.3390/ph6121543 PMC3873676

[11] Mosca DA, Hurst MA, So W, Viajar BS, Fujii CA, Falla TJ. IB-367, a protegrin peptide with in vitro and in vivo activities against the microflora associated with oral mucositis. Antimicrob Agents Chemother. 2000;44(7):1803–8. 10858334 10.1128/aac.44.7.1803-1808.2000 PMC89965

[12] Overhage J, Campisano A, Bains M, Torfs EC, Rehm BH, Hancock RE. Human host defense peptide LL-37 prevents bacterial biofilm formation. Infect Immun. 2008;76(9):4176–82. 10.1128/IAI.00318-08 18591225 PMC2519444

[13] Horswill AR, Stoodley P, Stewart PS, Parsek MR. The effect of the chemical, biological, and physical environment on quorum sensing in structured microbial communities. Anal Bioanal Chem. 2007;387(2):371–80. 10.1007/s00216-006-0720-y 17047948 PMC1797063

[14] Di Luca M, Maccari G, Maisetta G, Batoni G. BaAMPs: the database of biofilm-active antimicrobial peptides. Biofouling. 2015;31(2):193–9. 10.1080/08927014.2015.1021340 25760404

[15] Yomogida S, Nagaoka I, Yamashita T. Purification of the 11- and 5-kDa antibacterial polypeptides from guinea pig neutrophils. Arch Biochem Biophys. 1996;328(2):219–26. 10.1006/abbi.1996.0166 8644997

[16] Liu SP, Zhou L, Lakshminarayanan R, Beuerman RW. Multivalent Antimicrobial Peptides as Therapeutics: Design Principles and Structural Diversities. Int J Pept Res Ther. 2010;16(3):199–213. 10.1007/s10989-010-9230-z 20835389 PMC2931633

[17] Morrison GM, Rolfe M, Kilanowski FM, Cross SH, Dorin JR. Identification and characterization of a novel murine beta-defensin-related gene. Mamm Genome. 2002;13(8):445–51. 10.1007/s00335-002-3014-5 12226710

[18] Datta A, Kundu P, Bhunia A. Designing potent antimicrobial peptides by disulphide linked dimerization and N-terminal lipidation to increase antimicrobial activity and membrane perturbation: Structural insights into lipopolysaccharide binding. J Colloid Interface Sci. 2016;461:335–45. 10.1016/j.jcis.2015.09.036 26407061

[19] de la Fuente-Nunez C, Korolik V, Bains M, Nguyen U, Breidenstein EB, Horsman S, et al Inhibition of bacterial biofilm formation and swarming motility by a small synthetic cationic peptide. Antimicrob Agents Chemother. 2012;56(5):2696–704. 10.1128/AAC.00064-12 22354291 PMC3346644

[20] Wikler Matthew A. M, MBA, FIDSA, Jeff Alder P, Dudley Michael N. P, FIDSA, Eliopoulos George M. M, Ferraro Mary Jane P, MPH, Hardy Dwight J. P, et al Methods for Dilution Antimicrobial Susceptibility Tests for Bacteria That Grow Aerobically; Approved Standard—Ninth Edition M07-A9. Clinical and laboratory standards institute; 2012. Contract No.: 2.

[21] Cockerill Franklin R. I, MD, Wikler Matthew A. M, MBA, FIDSA, Jeff Alder P, Dudley Michael N. P, FIDSA, Eliopoulos George M. M, Ferraro Mary Jane P, MPH, et al Susceptibility Tests for Bacteria That Grow Aerobically; Approved Standard—Ninth Edition M07-A9 Clinical and laboratory standards institute; 2012. Contract No.: 2.

[22] Dean SN, Bishop BM, van Hoek ML. Susceptibility of Pseudomonas aeruginosa Biofilm to Alpha-Helical Peptides: D-enantiomer of LL-37. Front Microbiol. 2011;2:128 10.3389/fmicb.2011.00128 21772832 PMC3131519

[23] O'Toole GA. Microtiter Dish Biofilm Formation Assay. Journal of Visualized Experiments: JoVE. 2011(47):2437 10.3791/2437 21307833 PMC3182663

[24] Durham-Colleran MW, Verhoeven AB, van Hoek ML. Francisella novicida forms in vitro biofilms mediated by an orphan response regulator. Microb Ecol. 2010;59(3):457–65. 10.1007/s00248-009-9586-9 19763680

[25] Jordan GM, Yoshioka S, Terao T. The aggregation of bovine serum albumin in solution and in the solid state. J Pharm Pharmacol. 1994;46(3):182–5. 8027924 10.1111/j.2042-7158.1994.tb03774.x

[26] Yoshioka S, Aso Y, Izutsu K, Terao T. Aggregates formed during storage of beta-galactosidase in solution and in the freeze-dried state. Pharm Res. 1993;10(5):687–91. 8321832 10.1023/a:1018951530927

[27] Wu S-L, Leung D, Tretyakov L, Hu J, Guzzetta A, Wang YJ. The formation and mechanism of multimerization in a freeze-dried peptide. International Journal of Pharmaceutics. 2000;200(1):1–16. 10845681 10.1016/s0378-5173(99)00469-x

[28] Chandrasekhar S, Moorthy BS, Xie R, Topp EM. Thiol-Disulfide Exchange in Human Growth Hormone. Pharm Res. 2016;33(6):1370–82. 10.1007/s11095-016-1879-3 26887678 PMC4854756

[29] Nagant C, Pitts B, Nazmi K, Vandenbranden M, Bolscher JG, Stewart PS, et al Identification of peptides derived from the human antimicrobial peptide LL-37 active against biofilms formed by Pseudomonas aeruginosa using a library of truncated fragments. Antimicrob Agents Chemother. 2012;56(11):5698–708. 10.1128/AAC.00918-12 22908164 PMC3486595

[30] Paulsen VS, Blencke HM, Benincasa M, Haug T, Eksteen JJ, Styrvold OB, et al Structure-activity relationships of the antimicrobial peptide arasin 1—and mode of action studies of the N-terminal, proline-rich region. PLoS One. 2013;8(1):e53326 10.1371/journal.pone.0053326 23326415 PMC3543460

[31] Crusca E Jr., Rezende AA, Marchetto R, Mendes-Giannini MJ, Fontes W, Castro MS, et al Influence of N-terminus modifications on the biological activity, membrane interaction, and secondary structure of the antimicrobial peptide hylin-a1. Biopolymers. 2011;96(1):41–8. 10.1002/bip.21454 20560142

[32] Singh PK, Parsek MR, Greenberg EP, Welsh MJ. A component of innate immunity prevents bacterial biofilm development. Nature. 2002;417(6888):552–5. 10.1038/417552a 12037568

[33] Haisma EM, de Breij A, Chan H, van Dissel JT, Drijfhout JW, Hiemstra PS, et al LL-37-derived peptides eradicate multidrug-resistant Staphylococcus aureus from thermally wounded human skin equivalents. Antimicrob Agents Chemother. 2014;58(8):4411–9. 10.1128/AAC.02554-14 24841266 PMC4136056

[34] de la Fuente-Nunez C, Mansour SC, Wang Z, Jiang L, Breidenstein EB, Elliott M, et al Anti-Biofilm and Immunomodulatory Activities of Peptides That Inhibit Biofilms Formed by Pathogens Isolated from Cystic Fibrosis Patients. Antibiotics (Basel). 2014;3(4):509–26. 26221537 10.3390/antibiotics3040509 PMC4515429

[35] Reffuveille F, de la Fuente-Nunez C, Mansour S, Hancock RE. A broad-spectrum antibiofilm peptide enhances antibiotic action against bacterial biofilms. Antimicrob Agents Chemother. 2014;58(9):5363–71. 10.1128/AAC.03163-14 24982074 PMC4135845

[36] Gordon CA, Hodges NA, Marriott C. Antibiotic interaction and diffusion through alginate and exopolysaccharide of cystic fibrosis-derived Pseudomonas aeruginosa. J Antimicrob Chemother. 1988;22(5):667–74. 3145268 10.1093/jac/22.5.667

[37] Gulot E, Georges P, Brun A, Fontaine-Aupart MP, Bellon-Fontaine MN, Briandet R. Heterogeneity of diffusion inside microbial biofilms determined by fluorescence correlation spectroscopy under two-photon excitation. Photochem Photobiol. 2002;75(6):570–8. 12081317 10.1562/0031-8655(2002)075<0570:hodimb>2.0.co;2

[38] Shigeta M, Tanaka G, Komatsuzawa H, Sugai M, Suginaka H, Usui T. Permeation of antimicrobial agents through Pseudomonas aeruginosa biofilms: a simple method. Chemotherapy. 1997;43(5):340–5. 9309367 10.1159/000239587

[39] Begolo D, Erben E, Clayton C. Drug target identification using a trypanosome overexpression library. Antimicrob Agents Chemother. 2014;58(10):6260–4. 10.1128/AAC.03338-14 25049244 PMC4187942

