# Supplementary material for: Peptide modification results in the formation of a dimer with a 60-fold enhanced antimicrobial activity
Source: PLoS One. 2017 Mar 15;12(3):e0173783. doi: 10.1371/journal.pone.0173783 (PMC5351969; doi:10.1371/journal.pone.0173783)
Supplement: S1 Table — (DOCX) [file pone.0173783.s001.docx]

**S1 Table**. Inhibitory effect of the peptides on bacterial growth.^a^

| Bacteria (strain) | MIC (μg/mL) | | | |
| --- | --- | --- | --- | --- |
|  | **pep1037** | **cys-pep1037**^b^ | **cys-pep1037 dimer** | **mal-cys-pep1037** |
| *P. aeruginosa* (PA01) | 307 | 40 | 21 | 357 |
| *P. aeruginosa* (PA14) | 307 | 40 | 21 | 357 |
| *E. coli* (DH5α) | 76 | 10 | 5 | n/d |

^a^Identical results were obtained for all replicates.

^b^Different stock solutions of **cys-pep1037** (13 mg/mL) used for this experiment contained variable amounts of **cys-pep1037 dimer**.
