# Supplementary material for: Peptide modification results in the formation of a dimer with a 60-fold enhanced antimicrobial activity
Source: PLoS One. 2017 Mar 15;12(3):e0173783. doi: 10.1371/journal.pone.0173783 (PMC5351969; doi:10.1371/journal.pone.0173783)
Supplement: S1 Fig — (DOCX) [file pone.0173783.s002.docx]

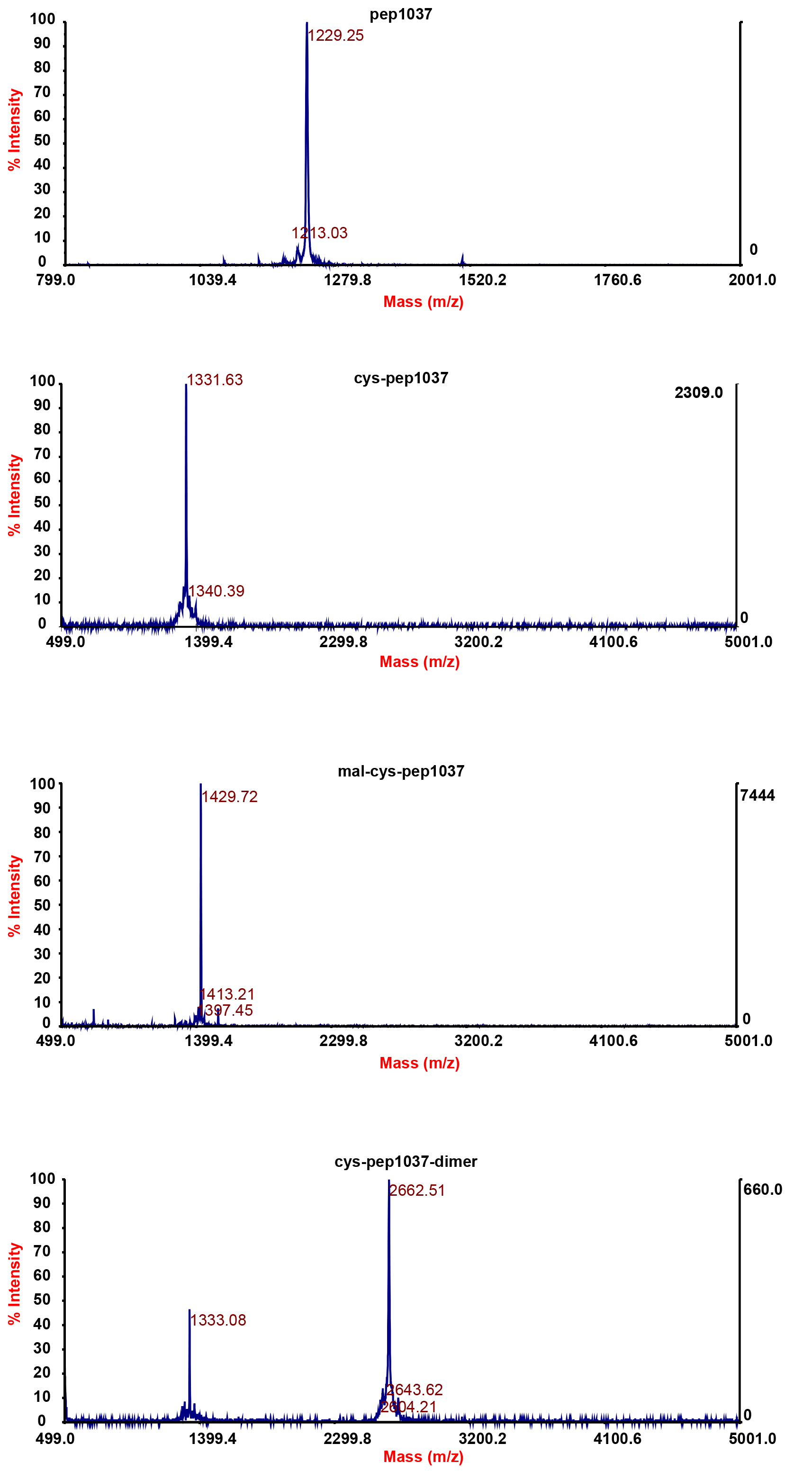


**S1 Fig.** Mass spectral data for all peptides (MALDI-TOF mass spectrometry in linear mode using [α](http://www.chemspider.com/Chemical-Structure.4485953.html)-cyanohydroxycinnamic acid as matrix).
